# Commentator Discussion: Out of the ice age: Preservation of cardiac allografts with a reusable 10 °C cooler

**DOI:** 10.1016/j.xjon.2024.09.013

**Published:** 2024-09-20

See Article page 197 in the October 2024 issue.

Presenter: Dr John Trahanas

**Dr Friedhelm Beyersdorf***(Frieberg, Germany)*. Yes. Thank you very much for this very interesting talk. I have 3 questions. You already mentioned the idea of having [inaudible] 10 °C is already kind of a long time ago. The whole world still puts most organs on ice if they are having not a finely perfused [inaudible]. And the question is why your data also showed that there are some significant differences in some laboratory data. And clinically you showed that there is a shorter length of stay in the hospital and a little bit of shorter stay in the intensive care unit even though it was significantly different as you have shown us. The pure numbers are, if I recall it correctly, about 3.02 or 3.2 or whatever. So, it's not really that much better even though the idea and the theory behind all that is very easily understood actually that at 0 °C—and you showed this very nice picture with the temperature that the mitochondria don’t work anymore very well. Free radicals are increased. So, my question to you is, first, why is there only a limited, let's say, clinical improvement? Even though as you showed us, the 6-hour group was obviously more or less significant, there was no control group. But why is it not taken up more?

**Figure:**
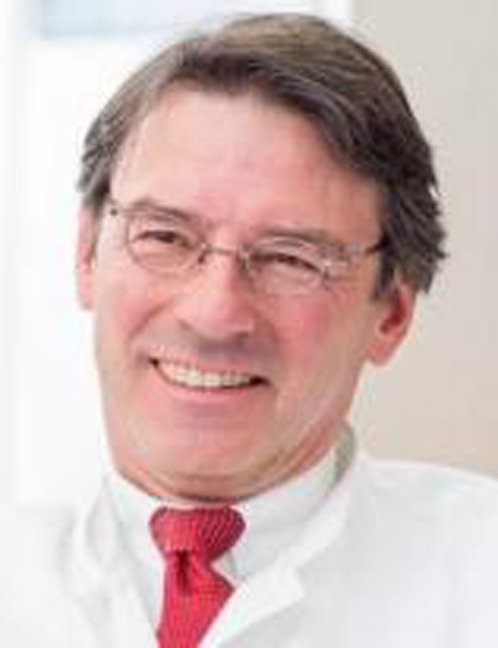

**Dr John Trahanas***(Nashville, Tenn)*. Yeah. Well, thank you for those questions. And to answer I think the last 1 first, in our initial analysis we did see that there seemed to be a difference in the length of stay, but in the larger cohorts, we actually saw that was not statistically significant. Why we did it? Well, as I mentioned, in lungs this has really been a transformative innovation. I mean now it's getting to the point where at our institution my elective coronary artery bypass grafts and aortic valve replacements are getting bumped in the morning because the lung transplant guys don't want to do their cases overnight. But luckily it works so well that now I tell them, “Do you know what? Just wait till I'm done,” and then do it in the afternoon. But really because it's worked so well in lungs that's what inspired us to try it in hearts. And we've been very pleased with the results that we've obtained with it. The mechanism by which it is protective—I am not an expert in subbiology. I'm sure Marcello or some of the Toronto guys could theorize as to why we think it most likely has to do with enabling cellular repair mechanisms and mitochondrial repair to function and not fully inhibiting those mechanisms. I wouldn't be the best person to answer that question.

**Figure:**
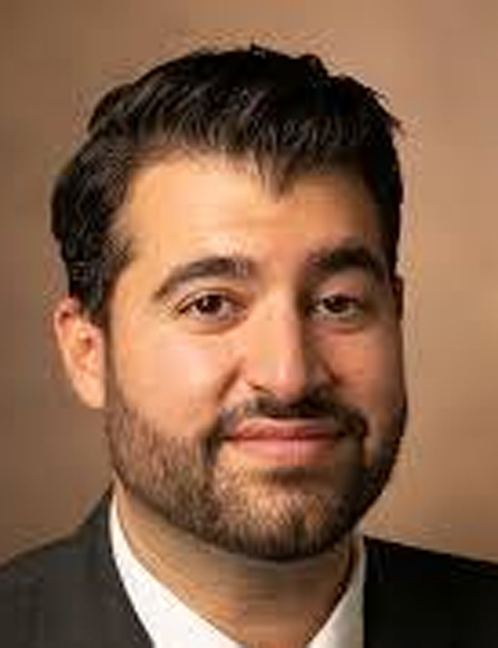

**Dr Beyersdorf**. Yeah. Thank you very much. My other question would be that the data on clinical outcome after a controlled hypothermic storage are still limited both for the lung and the heart even though the group from Toronto has definitely shown that there is room for tremendous improvement. For lung preservation, the trial on static ice storage was as controlled hypothermic storage is ongoing. And an update of the propensity-matched analysis of the guardian registry is awaited. Are you planning any of these kinds of studies for cardiac preservation using a 10 °C cooler? Because worldwide, I mean, there's still a certain percentage of primary graft failure. Whatever we do, even with the Organ Care System (TransMedics) or whatever we are planning to do and this of course has to be better. It has to be improved. So maybe this could be a study when whatever the results will be, finally more people take up this old/new technique.

**Dr Trahanas**. Absolutely. I agree. I think we do need to definitely study this more. I think we need to do this prospectively. We need to figure out long-term—not just for primary graft dysfunction, but what happens a year or what happens 2 years after these hearts are preserved at a higher temperature? Aren't you guys organizing said randomized study, and so hopefully that's something that will be coming soon, and we'll have those answers.

**Dr Beyersdorf**. Thank you very much.

**Dr Trahanas**. You're welcome.

**Dr Valluvan Jeevanandam***(Chicago, Ill)*. John, that was a very nice presentation, and congratulations on very low rates of primary graft dysfunction in both groups. But my question relates to how you managed the heart while you were implanting it. How we manage the heart valve while we're implanting it may influence the recovery after the ischemic period. Did you administer cardioplegia, for example? Were the 2 groups managed similarly during the implant process?

**Dr Trahanas**. So, there is a little bit, I think, of variation between the surgeons at our institution and the way that we reperfuse. I sewed the heart in, and I took the crossclamp off, and I did everything except the superior vena cava. I think Dr Shah likes to do a more controlled reperfusion, where he puts in the root vent and then slowly increases the temperature through the aortic root. But the percentage of the hearts done has been the same, both in the ice group by me and Dr Shah and others, and so I would hedge to guess that they're the same. But we didn't change our implant practices based on this method of preservation. They were the same in both.

**Dr Mohammed Quader***(Richmond, Va)*. Congratulations on your excellent outcomes. One thing that I have to say, the take-home message, is if you do things properly, there's nothing wrong with storing a heart in ice. And you have proved it. Okay? So, second thing, hypothermia, Dr Buckberg and a lot of work has been done on this, between cardioplegia and hypothermia, it's the hypothermia that is cardioprotective, not cardioplegia. So, for each 1 °C you drop, you decrease the metabolism by 5%. So, it doesn't make sense from that perspective that 10 would be better than 0. Right?

**Dr Trahanas**. Well, I think what we're starting to learn is that maybe it's not only about oxygen consumption and decrease in metabolism to completely 0, maybe that there is a protective effect of having some metabolism, and that's what I think we're starting to learn biologically in these organs.

**Dr Quader**. So just the last– [sorry?].

**Dr Trahanas**. And not to say ice is bad—ice has served us very well for 50 years—but at some point, we do need to come out of the ice age, or hopefully we will.

**Dr Quader**. Actually, ice had saved us to do lot of transplants. Don't discard it yet. So, the great work by Dr Bartley Griffith has shown that if you keep a heart at 4 °C, okay, it continues to accumulate lactate, and the pH goes down significantly. So that is at 4. So, it's very interesting, at the basic science, to see what happens to the heart when you store is at 10 °C. And how far are they going to push this? So, to say that we are going to increase the ischemia time by storing at 10 versus 4 versus 0, it just doesn't make sense from the cardioprotection side.

**Dr Trahanas**. It may not make sense, but I can tell you that the heart works when we take the clamp off. And I think we need to do more studies and figure out what the biologic mechanisms are. We don't have those answers yet, but—

**Dr Quader**. Thank you.

**Unidentified Speaker 2**. Congratulations, John. So, to answer the previous speaker, remember, Buckberg's studies was 4 °C cardioplegia that when it hit the heart, was 10 °C. So, the benefits were 10 °C, not 0 °C, which is what you're showing here. We show that actually, 20 °C in Toronto was better than 10 °C in hearts, and that was 20 years ago. But the caveat was it was a perfused heart at 20 °C. So, I think you have to compromise between perfusion to wash out the lactate versus the temperature you're storing at. We've now started using Traferox (Traferox Technologies Inc.) for hearts in Toronto, and we're very impressed with our results, so we share your enthusiasm. The 1 question I have for you, which you didn't really answer here, is those hearts that you subjected to 6 hours, how selective were you? Were you doing 50-year-old hearts that you knew were going to be 7 hours?

**Dr Trahanas**. I mean, that comes down to a case-by-case basis. I mean, obviously, we take everything into account. When we're looking at distance, we tend to try to make sure that it's a younger organ, if possible, not always. But we do consider the donor factors, the mechanism of death, the age, the travel time, the recipient. And it's a conglomerate of information, and in the end, we make a judgment call.

**Unidentified Speaker 3**. Have you looked at long-distance travel? Because what I find when you feel the hearts, if they travel on the Lear, they, when you go on a higher altitude, pressurize differently. They other being actually colder, right? So, the temperature may be 1 thing, but the damage to the mitochondrion, to the organelles, is probably why that super cold is not advantageous as opposed to 10 °C. And what we find is if you have ground transportation, you can maintain the temperature better. One of our colleagues is actually using a temperature recorder, and you can see that when you fly, the temperature actually goes down and goes much closer to 0 °C when you fly as opposed to when you have ground transportation. So, I think 10 °C is a great temperature. I think the key is trying to maintain it at 10 °C, which is the difficult component, especially when you're flying. So, I just wonder if you have any different techniques, you do when you fly as opposed to ground.

**Dr Trahanas**. No, we put it in there and we go. But as I showed, the temperature measured by the device stays pretty consistently very close to about 9 °C. And so, I'm not sure. Some of the group from Toronto may be able to comment if the device has a thermoregulatory mechanism related to altitude and pressure. But we have not seen wild swings in the temperature when we look at the readout from the device.

**Unidentified Speaker 3**. Great.

**Dr Trahanas**. Thank you.

**Unidentified Speaker 3**. Thank you.

[applause]

## Conflict of Interest Statement

The authors reported no conflicts of interest.

The *Journal* policy requires editors and reviewers to disclose conflicts of interest and to decline handling or reviewing manuscripts for which they may have a conflict of interest. The editors and reviewers of this article have no conflicts of interest.

